# Supplementary material for: Are carbon emissions trading and green financial instruments synergistic? -Comprehensive quantitative research based on content analysis
Source: PLoS One. 2024 Mar 7;19(3):e0298601. doi: 10.1371/journal.pone.0298601 (PMC10919601; doi:10.1371/journal.pone.0298601)
Supplement: S1 Data — (ZIP) [file pone.0298601.s001.zip › Data/地级市Z_F工作报告.zip!/║■▒▒/╢⌡╓▌╩╨/└┤╫╘╣1⁄2╓┌║┼í╛┬φ┐╦╩2╛▌═°í┐_2004.docx]

各位代表：

　　现在，我代表第四届人民政府，向大会报告过去五年的工作，对今后五年的发展思路和今年的工作提出建议，请予审议，并请市政协各位委员和列席人员提出意见。

　　一、过去五年的工作回顾

　　1999年到2003年，是鄂州跨世纪发展承前启后的重要时期。五年来，全市人民在省委、省政府和市委的正确领导下，高举邓小平理论伟大旗帜，深入贯彻党的十五大、十六大精神，认真实践“三个代表”重要思想，团结一心，奋发图强，圆满完成了市四届人大各次会议确定的各项奋斗目标。

　　综合经济实力不断增强。2003年，全市完成国内生产总值（预计，下同）125.7亿元,比1998年增长61.9%，年均递增10.1%。其中：完成第一产业增加值16.5亿元，比1998年增长19.4%，年均递增3.6%；完成第二产业增加值65.1亿元，比1998年增长97.6%，年均递增14.6%；完成第三产业增加值44.1亿元，比1998年增长1倍，年均递增15.3%。实现社会消费品零售总额48亿元，比1998年增长54.9%，年均递增9.1%；完成财政收入8.5亿元，比1998年增长71.7%，年均递增11.4%。

　　工业经济持续快速增长。2003年，全市规模以上工业企业实现产品销售收入135.7亿元,比1998年增长78.6%，年均递增12.5%；实现利税总额11.7亿元，比1998年增长67.1%，年均递增10.8%；2003年工业经济效益综合指数达148，居全省第一位。高新技术产业发展迅速，2003年完成增加值8.5亿元，比1998年增长2.5倍，年均递增28.5%。实施大集团战略取得显著成效，鄂钢集团5年共投入技改资金14亿元，2003年实现年产钢203万吨、销售收入48.3亿元、利税6.9亿元，比1998年分别增长90%、1.5倍、4.6倍，年均分别递增14.1%、20%、41.2% 。

　　农业结构调整成效显著。2003年，水产、畜禽、蔬菜、果木四大支柱产业实现增加值12.9亿元,占农业增加值的78.2%；水产品产量、畜禽出栏量、蔬菜产量五年年均分别递增10.4%、16.5%、13.5%。两条10公里水产走廊被定为省级优质水产品基地。建起了两个万头原（良）种猪场和10个万头猪场，牲猪生产被列为全省外贸出口基地和优质三元猪基地。油菜生产在全省率先实现“双低化”，被国家授予科技推广二等奖。燕矶、杨叶、临江、蒲团等4个万亩蔬菜基地被授予全省无公害蔬菜基地。退耕还林8万亩，“万树村”活动扎实推进，森林覆盖率达到14%，比1998年增加6个百分点。五年来，农业综合开发累计投入4609万元，改造中低产田13.7万亩。水利基础设施建设共完成投资7.8亿元，加固维修了长江干堤66.7公里、内湖堤防212公里和涵闸泵站310座，35座水库整险达标，我市被省政府授予长江堤防建设先进单位。

　　第三产业发展势头良好。通过引进国内知名商贸企业，发展超市、专卖店等新型流通业态，盘活了亚太广场、鄂州商场、鄂东商场等6家企业的存量资产。顺利完成明堂市场改建工程，兴建各类商品市场16个，成立各类农副产品流通组织56个，活跃了城乡市场。成功举办了三届武昌鱼国际文化旅游节和四届梁子湖捕鱼旅游节，我市荣获“中国优秀旅游城市”称号，对外形象和知名度得到提升。第三产业对GDP增长的贡献率达41.2%，拉动经济增长4.2个百分点。

　　各项改革深入推进。国企“两改”不断深化，列入市级改制计划的118家企业，基本完成改制任务113家，协议安置职工42776人，分别占总数的95.8%和95.3%。国企改革推进了民营经济快速发展，五年共发展个体工商户13931户、私营企业1515家，提供就业岗位47629个。积极推进国有资产管理体制改革，形成了三级营运、监督、管理体系。全面实施行政事业单位改革，市、区行政机构分别减少22.2%、11.8% ；行政编制分别减少28.7%、21.6% 。乡镇事业机构精减60%，编制精简25%；撤、并、转市直事业单位78家，精简编制1902个，分流安置职工2399人。二医院整体向民营业主出售取得成功。农村税费改革基本完成，全市农民负担总额减少4200万元，减幅率达49%，人平减负和亩平减负分别为48.5%、43.1%，我市被评为全省农村税费改革优秀单位。财政体制改革不断深化，全面推行部门预算、财会集中核算、政府采购、乡镇“零户统管”和“收支两条线”管理，财政体制进一步完善。国土资源管理得到加强，经营性土地全部实行市场挂牌交易或竞标拍卖。

　　开放引进取得新进展。2003年完成招商引资（含市外国内资金）1.8亿美元,比1998年增长2.3倍,年均递增26.9%；实际利用外资5021万美元,比1998年增长1.1倍,年均递增15.9% 。外贸出口总值完成2.31亿美元，比1998年增长87.8%，年均递增13.4% 。累计完成固定资产投资152.2亿元，年均递增7%；国债资金项目46个，到位资金13.5亿元。葛店高新技术开发区、花湖民营开发区、樊口工业园等8个经济园区发展势头良好。出口结构逐步优化，农副产品和机电产品出口有较大幅度增长；全市出口企业达33家，比1998年增长3.3倍。

　　财税金融平稳运行。2003年，财政收入质量明显提高，完成国税收入4.68亿元，地税收入2.92亿元，国地两税收入占财政收入的比重达到89.4%，比1998年提高了16个百分点。银行信用社支付风险得到化解，信贷结构不断优化，经营效益明显提高。2003年底各项存贷款余额分别为90.1亿元和73.7亿元，比1998年分别增加51.7亿元和26.6亿元，年均分别递增16.9%和9.2%。商业保险得到迅速发展，2003年各项保费收入1.6亿元，比1998年增长1.3倍，年均递增16.3%。

　　城市建设步伐加快。制定了《鄂州市城镇化发展战略》，完成了《鄂州市城市总体规划》修编，各区、乡镇和66%的村实施了规划管理。城市基础设施五年累计投资5.2亿元，新增城市道路55万平方米，扩大城区面积10平方公里。鄂黄大桥、凤凰广场、南浦虹桥、鄂州高中、广电大楼等标志性建筑为城市增添了新的亮点。洋澜湖综合治理成效明显,湖面面积由整治前的2300亩恢复到5009亩。完成城市污水处理一期工程，延伸供水管网61.7公里。城区新增园林绿地面积305亩，人均公共绿地面积达到8.8平方米。城市管理力度不断加大，市容市貌变化明显。小城镇建设完成投资2.4亿元,村镇基础设施得到较大改善,城镇化率达51.3%。全面实施环境保护“一控双达标”工程，投资7.2亿元完成环保治理项目40个，关停对环境造成污染的小工厂235家，梁子湖被批准为省级湿地保护区，我市被确定为全国生态示范试点城市。交通建设投入5.71亿元，鄂黄大桥一级公路连接线（鄂州大道）、三一六国道改造、樊湖公路改造、五丈港码头建设、梁子湖码头及航道疏浚等重大工程相继竣工，农村水泥路面完成543.4公里，基本实现村村通水泥路，我市被列为全省农村公路建设示范市。城乡电网改造累计投入6.3亿元，新建变电站6座，年供电量达到23.3亿千瓦时，比1998年增长60%，年均递增10%。五年共投入9.1亿元，用于通讯、信息设施建设，全市拥有程控电话17万门，移动电话14.2万部。广播电视网实现了无缝隙覆盖。我市被列为全省首批信息化试点城市。

　　发展环境不断改善。围绕转变职能，提高效率，先后取消、下放、合并和转移825项行政审批事项，减幅达47.8%；建立了行政服务中心，实行“一站式”办公和 “一条龙”服务。全面推行企业“宁静日”制度，加强市场监管，整顿经济秩序，努力营造公平竞争的市场环境。大力实施服务承诺制和责任追究制，政务公开度不断增大，政府各部门服务意识不断增强。打造“信用鄂州”取得成效，我市信用等级由D级跳升到B级。

　　群众生活水平逐步提高。2003年城镇人均可支配收入达到6910元，年均递增5.2%；农民人平纯收入2832元，年均递增3.9%。全市人均居住面积32.8平方米，比1998年增长2.6倍，年均递增29.2%；移民建镇使5236户居民安居乐业。社会保障工作进一步加强，“三条保障线”得到巩固，离退休人员养老金、下岗职工基本生活费按时足额发放，符合条件的城镇贫困居民，全部纳入了最低生活保障范围。全面落实再就业优惠政策，五年间先后安置城镇下岗失业人员73432人，城镇登记失业率为4.2%。重视扶贫帮困工作，五年开发扶贫项目117个；实施“福星工程”，改扩建福利院23所，农村五保户供养政策基本落实，生活得到妥善安排。

　　社会事业全面进步。五年来，共实施国家、省科技计划项目229个，引进外国专家76人次，解决技术难题85个，20家企业获省级高新技术企业称号，科技对经济增长的贡献率达43.6%。“普九”成果进一步巩固，教育改革不断深化，农村教育实行了以区为主的管理体制，教师工资发放实现了区级统筹；学校布局得到合理调整，中小学实行新课程改革；共投入1.77亿元，改建中小学危房11.4万平方米。群众文化体育活动蓬勃开展，“周周乐”广场文化深受市民喜爱，京剧《小凤》先后荣获湖北省“五个一工程”奖和国家文华奖。城乡医疗条件逐步改善，血吸虫病、地方病防治工作得到加强；以对人民群众高度负责的精神，扎扎实实开展了2003年的“非典”防治工作，保证了鄂州“一方净土”。平抑新生儿性别比失衡工作取得明显成效，人口出生率比省定责任目标年均低6.1个千分点，保持了全市低生育水平。安全生产逐步加强，特大安全事故得到控制。群众性精神文明创建活动蓬勃发展，市民文明程度不断提高。国防教育、征兵和民兵预备役工作不断加强，军政、军民关系进一步密切。我市多次被评为省级文明城市、卫生城市、园林城市、双拥模范城。统计、信访、对台、外事侨务、人防、民族宗教等各项工作取得新成绩，档案、气象、农机、老龄、残疾人保障等事业有了新的发展。

　　民主法制建设不断加强。自觉接受人大、政协的监督，五年共办理人大代表议案和建议、政协委员提案1050件，满意率达到98.8%。重大决策注重征求民主党派、工商联、无党派人士、人民团体、咨询研究部门和基层群众的意见，推进了政府决策的民主化。积极推进依法治市，加强政府法制建设，国家机关工作人员法制观念不断增强。领导干部廉洁自律、大案要案查处和源头治腐力度加大，审计、监察工作得到加强，廉政建设的各项规定得到较好的贯彻。“严打”整治斗争和“法轮功”练功人员的帮教转化工作成效明显，社会治安综合治理步入全国先进行列。全民法制宣传教育深入进行，“三五”普法经省验收合格，“四五”普法开局良好。

　　各位代表，过去的五年，是我市经济持续快速发展、综合实力显著增强的五年，是城乡面貌发生巨大变化、人民生活水平进一步提高的五年，是改革开放取得重大突破、经济发展活力不断增强的五年，是三个文明协调发展、各项社会事业全面进步的五年。回顾总结过去五年的政府工作，有四条基本经验值得在今后的工作中借鉴。一是坚持解放思想，实事求是，与时俱进，不断探索加快鄂州发展的新思路。五年来，我们始终以解放思想为先导，紧密结合鄂州实际，将工业作为加快鄂州发展的“牛鼻子”，以工业化带动产业化、城镇化、现代化，在发展思路上实现了由“工业立市”向“工业兴市”的转变，由传统农业向城效型农业的转变，由公有制为主体到公有、民营、外资和混合制经济竞相发展的转变。二是坚持以改革总揽全局，通过深化改革不断克服前进中的各种困难。五年来，我们先后通过实施以“两改”为主要内容的国企改革，一大批中小企业实现民有民营，企业活力明显增强；通过深入推进农村税费改革，全市农民人均负担大幅减轻，生产经营的积极性有了极大提高；通过实行财政体制综合改革，公共财政框架初步形成。三是坚持执政为民，始终把维护群众利益放在政府工作的首位，努力为广大人民群众办实事，办好事。五年来，我们始终牢固树立“执政为民”的思想，坚持把解决群众关心的热点难点问题作为政府工作的重要内容，集中人力、物力、财力，做好城乡弱势群体救助、下岗职工再就业、农村公路建设、农业基础设施建设、生态环境保护等工作，进一步密切了党和政府与人民群众的联系。四是坚持求真务实，努力转变政府职能，加强作风建设，优化经济发展环境。五年来，我们注重正确处理政府与企业的关系，通过深入开展“三个代表”重要思想学习教育活动，引导全市上下树立“政府创造环境，企业创造财富”的新观念，维护市场主体的合法权益。大力弘扬艰苦奋斗的优良作风，从思想教育和纪律约束入手，解决“门难进、脸难看、事难办”的“衙门”作风，政府各部门的工作作风不断好转。

　　各位代表，五年的成绩来之不易，凝聚着全市人民的智慧和心血。借此机会，我代表市人民政府向全体代表、政协委员，向全市各条战线的广大干部和群众、离退休老同志，向驻市的解放军、武警和消防官兵，向各民主党派、工商联、无党派人士、人民团体，向关心支持我市建设的港澳台同胞、海外侨胞、国际友人表示衷心的感谢！

　　在充分肯定成绩的同时，我们也清醒地看到，当前经济社会生活中还存在着不少矛盾和问题：思想解放不够，开放引进步伐还不快；市场主体培育不够，经济活力不足；农村经济发展不够，农民增收缓慢；提供就业岗位不够，下岗职工生活困难；区乡财源建设不够，市级财政偿债压力大；少数政府工作人员作风转变不够，不同程度地存在着形式主义、官僚主义现象，廉政建设和反腐败工作还需进一步加强。这些问题已引起我们的高度重视，今后将采取积极的措施，努力加以解决。

　　二、今后五年的工作建议

　　今后五年，是鄂州贯彻落实党的十六大精神，全面建设富裕型小康城市的重要时期，是一个必须紧紧抓住并且可以大有作为的重要战略机遇期。五年政府工作的指导思想是：高举邓小平理论伟大旗帜，全面贯彻“三个代表”重要思想，按照党的十六届三中全会提出的“五个统筹”要求，坚持以建设富裕型小康城市为目标，以广大人民群众根本利益为出发点，以发展为主题，以结构调整为主线，以改革开放为动力，继续实施“一化带三化”战略，解放思想、实事求是、与时俱进、开拓创新，切实加强社会主义物质文明、政治文明和精神文明建设，不断提高经济运行的质量和效益，不断培植经济发展的后劲，努力开创我市经济社会全面、协调、可持续发展的新局面。主要奋斗目标和任务是：

　　——进一步增强经济实力。到2008年，国内生产总值达到212亿元，年均递增11%；财政收入达到15亿元，年均递增12%；全社会固定资产投资75亿元，年均递增16%；外贸出口总额3.7亿美元，年均递增10%。力争经济发展速度和运行质量处于全省领先水平。

　　——进一步优化产业结构。到2008年，一、二、三次产业比重达到8:52:40。力争年税收过亿元的企业超过3家，过3000万元的企业超过8家，过1000万元的企业超过20家。建成全省无公害水产品生产和加工基地、畜禽原种生产和出口创汇基地。建成武汉城市圈的休闲度假首选地。

　　——进一步改善人民群众生活。到2008年，城镇居民人均可支配收入9247元，年均递增6%，农民人平纯收入3770元，年均递增5.8%。每年净增就业岗位1.5万个，城镇登记失业率控制在全省平均水平以下。城乡居民人均居住面积增加5平方米。森林覆盖率提高6个百分点，城市人均绿地面积增加4平方米。人口自然增长率控制在5‰以内，城镇化率达到56%。

　　从国内外经济发展格局和我市基本态势来看，加入世贸组织的积极效应逐步显现，区域经济一体化进程进一步加快，新一轮经济增长周期初见端倪，为加快发展提供了难得的机遇；我市经济体制改革的深化，市场化程度的提高，民营经济的快速发展，将不断增强加快发展的内生动力；通过二十多年的努力，我市改革开放和经济建设取得了巨大的成就，全市上下精神振奋、政通人和，为加快发展打下了坚实的思想和物质基础；特别是近年来，我市项目建设取得重大突破，一批新的经济增长点正在逐步形成，为加快发展提供了有力的支撑。我们必须进一步认清形势、坚定信心、把握机遇、同心同德、励精图治，在应对挑战中争取主动，在战胜困难中经受考验，在扬长避短中增创优势，在超越自我中寻求突破，以新的作为推进鄂州经济社会全面发展，确保上述奋斗目标和任务的圆满完成。不仅如此，我们还要以新的胆识，新的举措，新的追求，力争实现国内生产总值和财政收入翻一番。在今后五年的工作中，我们必须牢牢把握以下重点环节：

　　（一）立足鄂州市情，开拓发展新思路

　　加快鄂州发展，必须将国家的方针政策与鄂州的实际紧密结合起来，大胆探索，不断创新，理顺发展思路，明确发展措施。推进产业结构优化升级，走新型工业化道路。强化工业的主导地位，注重以信息化带动工业化，以工业化促进信息化，提升壮大冶金、建材、纺织服装、机电等传统产业，突破性发展生物医药、新材料、电子信息等高新技术产业，重点扶持一批有发展潜力的企业集团，培育一批国际国内的知名品牌，走出一条科技含量高、经济效益好、资源消耗低、环境污染少、人力资源优势得到充分发挥的新型工业化道路。推进农业产业化经营，走现代农业道路。按照“城郊型”农业定位，用抓工业的思路组织农业生产和经营，推进农业生产工厂化、种养模式立体化、产品标准化、经营产业化、市场国际化。加快结构调整步伐，切实做大做强水产、畜禽、蔬菜、果木四大支柱产业，提高我市农产品竞争力和农业综合效益。推进城镇化进程，走城乡协调发展道路。坚持城镇化建设与本地实际相结合，充分发挥区位优势和资源优势，强化城市规划管理，优先发展主城区，加快发展西部城区，重点发展中心镇，形成各具特色的城镇网络。坚持城镇化建设与产业化相结合，使城镇成为农村工业的小区，第三产业配套发展的载体，形成规模聚合效应。树立“依武而昌”的观念，形成全面对接、优势互补的新格局。推进科教兴市战略，走创新发展道路。加快建设以企业为主体、引进消化吸收和自主创新相结合的科技创新体系，加速新技术的推广应用，促进科技与经济相结合。深化教育体制改革，推进素质教育，优化教育结构，加快建立现代国民教育体系，努力建设全民学习、终身学习的学习型城市。坚持计划生育、保护环境和保护资源的基本国策，着力构建自然和谐的城镇人居环境、良性循环的农村生态环境，促进经济、社会和环境协调发展。

　　（二）不断创新体制，实现改革新突破

　　加快鄂州发展，必须不断深化改革，用改革的办法解决影响我市经济社会发展的深层次矛盾。在发展非公有制经济上取得新突破。以产权制度改革为核心，大力发展非公有制经济，在市场准入、上市融资、进出口贸易、政府采购、招标投标、项目申报、科技奖励、人才引进等方面给予公平待遇，推进所有制形式多元化。在完善市场体系上取得新突破。更大程度地发挥市场在资源配置中的基础性作用，发展资本市场，扩大企业融资渠道，鼓励并支持企业上市，着力解决中小企业发展资金不足的问题；发展劳动力市场，建立就业服务体系，合理配置劳动力资源；发展技术市场，促进科研成果的应用和转化，加快科技长入经济的步伐。在完善社会保障制度上取得新突破。健全独立于企事业单位之外、资金来源多元化、保障制度规范化、管理服务社会化的社会保障体系，完善对低收入者的救助制度。发展城乡社会救济、社会福利及慈善事业，做到幼有所育、少有所学、老有所养、病有所医、困有所帮。

　　（三）扩大招商引资，再创开放新局面

　　加快鄂州发展，必须把招商引资作为经济工作的重中之重，在更大范围、更高层次、更宽领域推进对外开放。在提高开放水平方面取得新进展。进一步加大招商引资力度，引进项目力求引大、引强、引新，引进资金力求引多、引实、引快。整合资源，强化规划，不断提高各类开发区和工业园区建设水平，充分发挥开发区的示范带头作用。大力实施以质取胜、多元发展和“走出去”战略，不断提高我市经济的外向度。在扩大开放领域方面取得新进展。加大工业、农业和基础设施吸收外资力度，扩大商贸流通、金融、旅游、中介等领域的对外开放。充分发挥我市毗邻武汉的区位优势，抓住建设武汉城市圈的机遇，不断提升鄂州与武汉的经济合作水平。在优化开放环境方面取得新进展。进一步改善法制环境、政策环境、市场环境、信用环境、生态环境，不断增强综合环境竞争力，把鄂州建成全省投资成本最低、投资效益最佳、服务质量最优、发展环境最好、对外商吸引力最强的“磁场”。

　　（四）切实转变职能，塑造政府新形象

　　加快鄂州发展，必须努力抓好政府的自身建设，塑造“为民、务实、清廉”的政府形象。努力建设学习型政府。深入学习邓小平理论和“三个代表”重要思想，学习市场经济、金融、科技、法律、历史知识，不断提高公务员队伍思想水平、政治素质和业务能力，以适应时代发展的要求。努力建设民主型政府。接受人大及其常委会的法律监督，及时报告工作，落实各项决议、决定。接受政协的民主监督，听取各民主党派、工商联及各人民团体的意见，认真办理人大代表建议和政协委员提案。建立重大事项社会公示制度和社会听证制度，完善专家咨询制度，推进电子政务，拓宽人民群众参与管理经济、文化和社会事务的渠道，实现政府决策的科学化、民主化。努力建设服务型政府。强化宗旨观念和公仆意识，进一步转变职能，提高政府的社会管理和公共服务水平。继续办好行政服务中心，规范发展行业协会、商会等社会中介组织。坚持依法行政，强化行政执法监督和责任追究。切实解决群众关心的热点难点问题，努力为人民群众办实事、办好事。努力建设务实型政府。大力弘扬求真务实的作风，脚踏实地，注重实效，坚决反对形式主义、官僚主义，克服弄虚作假、好大喜功、急功近利等行为。进一步转变作风，大力精简会议和文件，深入基层，深入群众，狠抓各种决策和工作部署的督办落实。努力建设廉洁型政府。坚持艰苦创业，勤俭办一切事业，完善公共财政管理体制，狠刹各种奢侈浪费之风。严格执行廉洁自律各项规定，坚持不懈地抓好廉政建设和反腐败工作，全面推行干部经济责任审计，从严治政，标本兼治，从源头上防止腐败。

　　三、2004年工作安排

　　2004年是新一届政府的开局之年。五年看头年，头年看起步，做好今年的政府工作，意义重大。今年经济社会发展的预期目标是：国内生产总值增长11%，达到139.5亿元；财政收入增长12%，达到9.5亿元；全社会固定资产投资增长16%，达到43.5亿元；社会消费品零售总额增长10%，达到52.8亿元；外贸出口增长12%，达到2.6亿美元；实际利用外资增长10%，达到5523万美元；城镇居民人均可支配收入增长6%，达到7325元；农民人平纯收入增长6%，达到3002元。为了完成上述目标，今年要重点抓好以下几项工作：

　　（一）以推进新型工业化为先导，提高经济运行质量和效益

　　始终把工业发展的重点放在提高经济运行质量和效益上，努力形成布局合理、结构优化、产业特色优势明显、核心竞争力强的新型工业经济发展格局。

　　加快实施大公司大集团战略。支持鄂钢、程潮铁矿、鄂州电厂等中央、省属企业做大做强，进一步凸现对鄂州经济的支撑地位。重点扶持多佳、武昌鱼、顾地塑胶、波尔亚太、化研院集团、科益药业、科氏沥青、枫树、吴城钢铁、世纪新峰等骨干企业，通过上市、重组等多种途径，不断扩张生产规模，催生裂变新的关联产业和企业，形成一批有竞争力的强势企业群。充分发挥工业园区的产业聚集作用，葛店高新区按照“百亿园区”的建设定位，重点建设生物技术与新医药工业园，将“中国药谷”打造成全省一流、全国知名的品牌。花湖、小桥、樊口、新庙、燕矶、华容、梁子湖等园区继续坚持以特兴园的发展思路，不断提高竞争实力。

　　大力发展中小企业。积极发展与中央省属企业的配套服务产业，延伸产业链条，促使我市相关中小企业与中央、省企业良性互动，实现“双赢”。采取政策引导、信息引导和服务引导的办法，推动各类工业企业由粗放型向集约型转变，由“小而全”向“小而强”转变，由家庭式管理向现代化管理转变，提升中小企业素质。

　　不断增强企业核心竞争力。加快企业技术改造步伐，积极鼓励和支持冶金、服装、建材、机电等传统产业运用高新技术、环保技术、节能技术，改造提高工艺和装备水平，淘汰技术水平低、役龄超期的工艺装备，实现技术跨越，促进产业升级。加快工业信息化进程，运用信息技术推进生产过程自动化、控制智能化，抓好鄂重等7家企业的信息化改造，引导扶持科益药业、春天药业、人福科技等10家企业的信息化应用。大力实施精品名牌战略。采取得力措施，促进优质资产向优势企业和名牌产品聚集，认真实施重大新产品开发专项滚动计划和产品储备计划，不断提高产品质量，加强广告宣传，运用现代营销策略，提高产品的市场占有率。

　　（二）以结构调整为主线，实现农业增效农民增收

　　进一步加大农业结构调整力度，推进农业产业化经营，促进水产、畜禽、蔬菜、果木四大支柱产业快速发展。

　　加快农业基地建设。水产业重点培育武昌鱼、河蟹、珍珠、红尾鱼等名优品种，积极创建首强水产市、10强水产乡镇、20强水产村，全面推进50万亩无公害水产基地建设，形成独具特色的水产走廊。畜禽业进一步推进优质畜禽产品规模化养殖，力争建成12个万头优质三元猪场、20万只绿壳蛋鸡和150万只优质水禽三大养殖带，积极争取省政府和相关部门的支持，新建湖北种牛场和种鹅场。蔬菜业按照无毒、无残留、无污染的要求，加快建设8个万亩无公害蔬菜基地。果木业重点建设东佛苗圃和红莲湖花卉等10个花卉苗木基地，加快10万亩胡柚基地建设步伐，确保今年新增胡柚种植面积3万亩。继续开展“万树村”创建活动，加快实施退耕还林工程。

大力发展龙头企业。按照“高、大、外、新”的要求，选择和培育一批成长性好、潜力大的龙头企业，在资金、技术、人才等方面给予重点扶持，进一步增强其市场竞争力和辐射带动力。市级重点扶持武昌鱼公司、嘉禾粮油公司、湖北原种猪场等骨干企业。各区重点培植3—5家对本地农产品加工转化增值大的企业。

　　努力提高农产品市场占有率。健全农产品质量标准和质量安全监测检测体系，加强对农产品产前、产中、产后的全程控制，严禁使用高毒、高残留农药，实行无公害生产、无公害加工、无公害流通。大力发展优质农产品，重点推广优质水稻、优质水产品、优质畜禽、优质蔬菜、优质果木等8个系列60多个优良品种，加快优质常规稻良种繁育基地建设，不断提高全市农产品优质率。切实保护农业耕地，努力提高粮食复种指数，深化粮食流通体制改革，注意保护农民种粮积极性。加快建设城东蔬菜批发市场，使我市成为鄂东南农副产品集散地。加强农业科技队伍建设，提高农业科技和信息服务水平。

　　加大农业基础设施投入。做好长江干堤整险加固煞尾工作，力争将粑铺大堤、昌大堤的主要堤段建成全省一流堤防。抓好病险水库的整治，加快抗旱、排涝设施建设。积极推进农业综合开发，全面实施农村“五改二建”工程（改水、改路、改厕、改圈、改灶、建庭院经济、建生态家园），不断改善农业生产条件和农民生活环境。切实做好农村劳动力输出的组织、管理和培训工作，鼓励引导农民进城务工经商，大力发展打工经济。

　　（三）以改革开放为动力，努力增强经济发展后劲

　　切实抓好以制度创新为重点的各项改革，扩大对外开放，加快项目建设，不断增创我市发展新优势。

　　加大体制改革力度。继续推进国企“两改”，大力发展混合所有制经济，培育新的市场主体，积极构建“归属清晰、权责明确、保护严格、流转顺畅”的现代产权制度。完善市级国有资产监管机制，按照管事、管人与管资产相结合的原则，理顺国有资产管理、监督、营运体制。继续深化农村改革，在稳定家庭土地承包经营的基础上，按照“依法、自愿、有偿、规范”的原则，推进土地合理流转，发展多种形式的土地适度规模经营。巩固农村税费改革成果，确保农民负担不反弹。继续加快政府机构改革，按照统一、精简、效能的原则，调整市政府职能机构设置，重点理顺综合经济部门职能；按照积极、稳妥的方针，抓好区级机构精减和乡镇综合配套改革，加强农村基层政权建设；按照政企分开的要求，完成长港农场的体制改革。继续推进社会事业单位改革，公益性事业单位推行内部人事、分配和用工制度改革，优化运行机制；经营性事业单位进一步加大产权制度改革力度，支持民营企业收购、参股经营性事业单位。

　　加大招商引资力度。不断创新招商引资方式，积极探索招商引资工作企业化、专业化运作的新路子，采取网上招商、以商招商、委托招商、园区招商、组团招商等多种形式，使我市招商引资工作再上一个新台阶。大力支持各类企业扩大加工贸易、自营出口和代理出口，在巩固港澳台、东南亚、日本等出口市场的基础上，大力拓展欧、美、非、中东等新兴出口市场；在保持纺织、服装等优势产品出口的同时，扩大机电和农副产品出口份额。

　　加大项目建设力度。千方百计增加投入，力争项目建设的数量和质量取得新突破。今年计划安排重点建设项目50个，突出抓好鄂钢三项技改工程、武钢500万吨球团矿厂、世纪新峰400万吨水泥一期工程等10个项目；确保顾地墙体涂料、扬子江药业、武昌鱼生化制药等10个项目的开工建设；加快鸿泰钢铁、浩信药业、健身药业、天然气工程、金刚石刀具等项目的建设步伐；加快鄂钢易地扩改、鄂州电厂二期改造等一批重大项目的进程。继续做好争取国债工作，力争凤山垃圾处理场、葛店开发区污水处理、区乡公路、退耕还林、社会发展等项目得到国债资金的支持。

　　加大财税金融工作力度。围绕推进新型工业化、农业产业化和高新技术产业化，发展县域经济，加强财源建设，各区重点培植3—5家产品竞争力强、市场占有率高、经济发展后劲足、税收过千万元的骨干企业；各乡镇重点培植3—5家税收过百万元的企业，壮大区、乡镇财政实力。继续深化财政体制改革，加强对财政收支的审计监管，提高对公共需要的保障能力，确保工资发放、社会保障、科技教育、扶贫支农等事关群众生活和社会稳定的支出。全面推行国库集中收付、部门预算，严格实行收支两条线，扩大政府采购范围，提高采购透明度。加强税收征管，强化税收稽查，坚决打击各种“偷、逃、抗、骗”税行为。认真抓好投融资体制改革，调整优化信贷结构，加强信用环境建设，密切银企、银农、银贸合作。促进金融部门进一步改善服务，认真落实对中小企业、民营企业、农业和农民的信贷支持措施，加大农村信贷投放力度。

　　（四）以推进城镇化建设为重点，统筹城乡经济社会发展

　　加快构建以主城区为中心，以西部城区、花湖、梁子湖三个组团为支撑的城镇发展新格局。

　　加强城市规划管理。坚持科学规划、合理布局、突出重点、体现特色的原则，抓紧城市总体规划的调整和市域城镇体系的编制。积极推行控制性详规管理制度，严格执行城镇“四线”即红线（道路）、绿线（园林绿化、山体）、紫线（文物古迹、历史文化名城、风景名胜区）、蓝线（江河、湖泊、湿地）管制制度。认真抓好城镇规划的实施，所有建设用地必须符合规划要求，对各类违章建筑要坚决拆除，确保城市建设步入规范化轨道。

　　加快城区基础设施建设。今年城市建设的重点是实施“一湖五桥九路”建设。继续实施洋澜湖环湖护岸、道路、绿化工程；完成月河桥维修加固工程，启动樊口大桥工程，兴建三座铁路专用立交桥；完成江碧路泽林至三民巷段改造及配套工程，完成滨江大道南浦路至油厂段道路工程，完成滨湖东路、滨湖南路路基及管网工程，完成凤凰路、洋澜路至寿昌大道延伸工程，完成古楼中街等老城区道路改造工程。兴建泽林至程潮进矿公路，兴建武钢球团矿铁路专用线。

　　加大小城镇建设力度。按照城市发展的总体规划，抓紧西部以葛店开发区为龙头的高新工业城镇群、东部以花湖开发区为龙头的民营工业城镇群、南部以梁子湖为中心的生态旅游城镇群的建设。加快乡村公路建设步伐，力争完成油路改造75.7公里，二级以上路基13.3公里、路面24.7公里，新建农村公路100公里，做好鄂黄大桥连接线至葛店的一级公路工程的前期工作，启动鄂州汽车客运中心建设。

　　大力发展第三产业。进一步加大各类社会中介服务机构的培育力度，充分发挥其在服务市场主体、配置市场资源和繁荣市场经济中的重要作用。进一步加大市场建设和管理的力度，整顿和规范市场经济秩序，继续发展连锁经营、物流配送、电子商务，积极支持超市、专卖店、品牌商区等各类流通业的发展，探索网上销售、远程交易等营销方式。进一步加大市场开拓力度，拓宽消费领域，培育新的消费热点，确保全市社会商品零售总额的持续增长。全面激活房地产市场，加快公房入市步伐，规范物业管理，培育、鼓励居民住房消费。进一步加大旅游产业发展力度，加快红莲湖综合开发、梁子岛基础设施建设步伐，积极开拓国内外客源市场，不断提高旅游接待水平和服务质量。

　　（五）以科教创新为支撑，促进各项社会事业发展

　　牢固树立科学技术是第一生产力的观念，大力实施科教兴市战略，高度重视人才工作，为富民强市奠定坚实的人才基础。

　　加快科技创新步伐。围绕产业升级，抓好科技开发、技术引进和成果转化。突出抓好生物医药、电子信息、新材料为主导的高新技术产业发展，确保3家高新技术企业、10项高新产品得到省级以上认定，力争高新技术产值比上年增长20%。注重技术开发，充分发挥企业在技术创新中的主体作用，形成一批具有自主知识产权的科技成果。建立风险投资机制，推进知识资本、金融资本、产业资本与民间资本的融合。牢固树立人才是第一资源的观念，坚持以人为本，尊重劳动，尊重知识，尊重人才，尊重创造。建立健全人才培养、引进、使用、激励机制，吸引各类人才来鄂州投资创业，施展才华。

　　大力发展教育事业。切实加强基础教育，巩固“普九”成果，严格控制辍学率，小学适龄儿童入学率保持100%，初中入学率达到98%以上，基本消除青壮年文盲。扩大高中阶段教育，加快发展高等教育，促进鄂州大学早日建成本科院校。积极发展职业教育、社区教育和现代网络教育。深化教育改革，鼓励社会力量办学，推进学校布局调整，大力改善办学条件，加强师资队伍建设，切实提高教育质量。

　　加强精神文明建设。认真贯彻公民道德建设实施纲要，弘扬爱国主义精神，以为人民服务为核心，以集体主义为原则，以诚实守信为重点，加强社会公德、职业道德和家庭美德教育。重视科普基础设施建设，加强科普宣传，破除封建迷信，倡导文明健康的生活方式。积极开展乡镇街文化创建活动，大力开展群众性体育活动，全面启动“全民健身朝阳工程”。广泛开展群众性精神文明创建活动，提高城乡文明程度。

　　加快社会事业发展。加强人口与计划生育工作，稳定低生育水平，采取综合措施平抑新生婴儿性别比。加强资源保护，合理开发和节约使用各种自然资源。加强环境监控和污染治理，全面推进生态示范市建设。坚持预防为主、防治结合的方针，提高应对突发公共卫生事件的能力，推进农村新型合作医疗改革试点工作进程。加强全民国防教育，积极做好民兵预备役和“双拥”工作。认真做好广播电视、对台、外事侨务、人防、民族宗教、老龄、残疾人保障等各项工作。

　　（六）以完善社会保障体系为着力点，不断改善人民群众生活

　　从改革、发展、稳定的大局出发，认真解决事关群众生活的突出问题，让人民群众在经济发展中得到更多实惠。

　　切实抓好再就业工作。认真落实再就业的扶持政策，改善就业和创业环境，拓展就业渠道，广开就业门路，建立以劳动者自主就业为主导，以市场调节就业为基础，以政府促进就业为动力的新机制。通过积极发展社区服务、商贸流通、餐饮等劳动密集型服务业，多层次、多领域增加就业岗位。加大再就业援助力度，确保税费减免、小额担保贷款等扶持政策落实到位。健全劳动监察、劳动争议调解和仲裁机制，保障劳动者的合法权益。

　　完善社会保障体系。继续扩大养老、失业、医疗、工伤和生育保险覆盖面，加强社会保障资金的监管，积极探索多渠道筹措社会保障资金的有效途径。进一步规范城市居民最低生活保障工作，把符合条件的城镇居民纳入低保范围，切实做到应保尽保。加大贫困乡村扶持力度，完善扶贫帮困机制，积极做好农村特困户社会救助工作。发展社会福利事业，加快建设农村“福星”工程，确保农村“五保”人员集中供养率达到85%以上。

　　切实维护社会稳定。做好群众来信来访工作，建立和健全社会矛盾纠纷排查调处工作机制，积极化解人民内部矛盾，妥善处理各类突发性群体事件。强化社会治安综合治理，超前防范和严厉打击各种违法犯罪活动，大力开展安全文明社区创建活动，加强社会治安防控体系建设。深入开展普法教育，不断提高公民特别是国家机关工作人员的法律素质。强化安全生产，消除各种重大事故隐患，确保人民生命财产安全。

　　各位代表，回首五年成就令人鼓舞，展望未来发展催人奋进。让我们紧密团结在以胡锦涛同志为总书记的党中央周围，高举邓小平理论伟大旗帜，全面贯彻“三个代表”重要思想，认真贯彻落实党的十六大和十六届三中全会精神，在省委、省政府和市委的领导下，万众一心，奋发图强，开拓创新，把鄂州改革开放和现代化建设事业不断推向前进。
